# Multi-annual spectral data of Chardonnay grapevine leaves presenting yellows diseases and confounding symptoms

**DOI:** 10.1038/s41597-025-06080-8

**Published:** 2025-12-17

**Authors:** Shurong Zhang, Eric Perrin, Valeriu Vrabie, Alban Goupil, Marie-Laure Panon

**Affiliations:** 1 Université de Reims Champagne-Ardenne, CReSTIC, 51100 Reims, France; 2 Comité Champagne, 5 Rue Henri Martin, 51200 Épernay, France

**Keywords:** Agriculture, Environmental impact

## Abstract

Grapevine yellows, a group of serious diseases affecting grapevines, have spread across grape-growing regions and represent a threat to vineyards, necessitating containment strategies. The identification of these diseases, particularly in the Chardonnay variety, remains a challenging and labor-intensive task. In this study, we present a comprehensive spectral dataset collected over five years from 700 grapevine leaves per year. The dataset includes leaves affected by yellows disease, healthy leaves as well as three additional classes consisting of leaves with visually similar symptoms, ensuring a diverse and representative sample. Spectral data were acquired under controlled laboratory conditions using a Labspec® 4i spectrometer, covering the spectral range from 350 nm to 2500 nm on grapevine leaves from areas of the experimental site of Comité Champagne. To facilitate further research, we performed basics analyses, including calculation of averaged spectra and principal component analysis, to explore spectral variations among different classes. This publicly available dataset serves as a valuable resource for researchers aiming to develop advanced techniques for robust detection and classification of yellows diseases.

## Background & Summary

Grapevine yellows diseases are caused by phytoplasmas. In Europe, the main grapevine yellows are Flavescence dorée (FD) and Bois noir (BN), which are associated with Flavescence dorée phytoplasma and ‘Candidatus Phytoplasma solani’, respectively. FD is one of the most devastating threats to viticulture, severely affecting all grape varieties with symptoms including leaf yellowing and rolling, a thickening of the leaves, shoots remaining green or partially lignify, reduced vigor, incomplete grape ripening, and eventual vine death. In addition to direct loss of yield and fruit quality, FD imposes a significant economic burden due to mandatory quarantine measures and costly replanting of affected vines^1^. Since there is no preventive treatment for this disease, the detected vines are commonly pulled out to prevent exponentially spreading to other vines. Indeed, vines can show symptoms at least one year after their infection, but have been an infectious source for the surrounding vines for one year already. Thus, early and efficient detection is critical to disease management, allowing targeted interventions such as the removal of infected vines to limit the spread of the disease within the vineyard.

Chardonnay, renowned for its versatility and global importance in wine production, is particularly susceptible to FD and shows several confounding symptoms, notably Leafroll (a viral disease), various types of discoloration, and to a lesser extent, Esca disease, a wood disease caused by a complex of fungi. Traditional visual inspections are resource intensive and often fail to distinguish these overlapping symptoms, especially for Chardonnay, highlighting the need for more efficient and scalable approaches to disease detection. In addition to FD, another yellows disease affecting grapevine is BN. Because FD and BN induce very similar syndromes at the leaf level, they are difficult to differentiate visually in the field and typically require PCR (Polymerase Chain Reaction) tests for distinction. In this work, FD and BN are grouped together under the category of yellows diseases, following Tessitori *et al*.^2^, as both pathogens produce comparable characteristic symptoms such as vein-limited yellowing of Chardonnay leaves with downward curling of the leaf blades into a triangular shape toward the end of the growing season, necrosis, poor shoot lignification, inflorescence abortion, and shriveled bunches later in the season.

Recent advances in precision agriculture and digital technologies have introduced promising alternatives for the detection of plant diseases. Techniques such as near-infrared spectroscopy and hyperspectral imaging can detect subtle physiological and biochemical changes associated with infection. By capturing spectral data over a wide range of narrow bands, these methods provide rich information that can be used to differentiate between healthy and diseased plants. Several studies have demonstrated the effectiveness of spectral analysis for disease detection. For example, Naidu *et al*.^3^ used spectral data to distinguish healthy grapevine leaves from those affected by leafroll disease. Extending this work, Hou *et al*.^4^ classified the disease into three stages according to severity, illustrating the potential of spectral techniques to monitor disease progression. Similarly, Owomugisha *et al*.^5^ explored the integration of spectral data with prototype learning algorithms to improve early disease detection and facilitate timely intervention strategies. In addition to grapevines, spectral analysis has been applied to other crops. Belasque *et al*.^6^ used laser-induced fluorescence spectroscopy to detect mechanical and disease-related stresses in citrus plants. This study underscores the versatility of spectral techniques in identifying plant health problems in a variety of agricultural settings.

Similar approaches have been explored in the context of FD detection. Al-Saddik *et al*.^7^ used spectral measurements of grapevine leaves to identify FD-specific signatures. Recent advances have demonstrated the potential of hyperspectral imaging for the detection of FD in grapevines. For example, the PhenoTruckAI project by Thielert *et al*.^8^ integrated hyperspectral screening and molecular diagnostics in a mobile laboratory to distinguish between FD and BN infections with high precision. Similarly, a study conducted by Carli *et al*.^9^ in Tuscany demonstrated the effectiveness of hyperspectral data in detecting early symptoms of FD and monitoring disease progression at the leaf level, with promising precision even before visible symptoms appear. However, the high-dimensionality of spectra and hyperspectral images poses significant challenges, including increased computational complexity and the risk of overfitting of supervised classification models. To overcome these challenges, dimensionality reduction techniques, such as the selection of optimal wavelengths or bands, or the use of spectral disease indices (SDIs), are often employed. These methods focus on using a limited number of spectral bands that are most informative for disease detection, simplifying the analysis. Note that pre-processing steps, including normalization techniques, have been shown to improve the consistency and interpretability of spectral data. Furthermore, the construction of suitable multispectral cameras with a limited number of optimal wavelengths or spectral bands might allow large-scale detection, thus facilitating vineyard surveys. However, the selected wavelengths or spectral bands must be robust and not subject to interference linked in particular to phytosanitary treatments or to changes linked to the weather conditions which depend on the years.

Based on these findings, the dataset presented thereafter focuses on the detection of yellows diseases, FD and BN, in Chardonnay vineyards using spectral reflectance data. It is important to note that yellows diseases in Chardonnay vineyards must be distinguished from similar symptoms. For this reason, in our acquisition dataset, we specifically collected leaves with yellows symptoms, commonly referred to as “jaundice” in French. As a result, all leaves with yellows symptoms should be detected as accurately as possible. To achieve this goal, spectral data was collected using a Labspec® 4i spectrometer under controlled laboratory conditions. Acquisitions were made on healthy leaves and leaves with jaundice, as well as on other visually confounding symptoms, Leafroll, Discoloration, and Esca, from different areas of the Comité Champagne experimental site over a 5-year period from 2020 to 2024. A subset of this dataset has been already used in our previous studies to identify the most discriminative wavelengths and spectral bands for jaundice detection in datasets collected over several years^10,11^. A key aspect of our analysis was to evaluate the robustness of these discriminative bands across different years so that they do not depend on environmental factors such as climate variations, humidity fluctuations, and other external influences that may affect spectral signatures. For practical use, it is essential that yellows disease detection models remain effective under varying environmental conditions and over multiple years. Robust results should be insensitive to inter-annual variability will ensure scalability and long-term applicability in real-world vineyard management.

The dataset presented below has been made publicly available to the research community to encourage researchers to use it to develop new algorithms or advance methods for robust detection of the yellow diseases. By providing access to this dataset, we aim to foster further research in the field of feature selection and extraction, and more broadly in the field of spectral analysis and precision agriculture. In this paper, we provide a detailed explanation of the data acquisition process for each year’s collection. To facilitate further research, we performed basics analyses, including a pre-processing technique to normalize the raw spectral data, the calculation of averaged spectra and an analysis using the principal component analysis (PCA). Indeeed, to better understand the spectral variations, we calculated the average spectra per class and per year, which allowed us to examine the similarities between classes and between different collection years. We performed PCA on the entire dataset to identify underlying patterns and trends by class and year. To facilitate the use of our dataset, we have included detailed usage notes to guide researchers on how to effectively manipulate the data.

This dataset can offer significant advantages for research and practical applications, enabling further analysis, classification, or comparison across years, zones, positions, and classes. It provides spectral data that comprehensively cover a wide range of grapevine symptoms for Chardonnay grapevine, including healthy and other confounding symptom diseased leaves.

The availability of this comprehensive dataset enables the development and validation of innovative analytical methods tailored to the specific challenges of viticulture. It could be used, for example, to explore the potential of spectral analysis to detect different symptoms and identify potential confounding factors. It could also be used to minimize environmental variability and improve the reproducibility of results from one acquisition year to another. It opens avenues for further research, such as exploring new alternative dimensionality reduction methods, feature selection or extraction methods. For example, it could help identify the most discriminating wavelengths or spectral bands, regardless of the acquisition year or region, or for the development of vegetation indices tailored for improved detection of jaundice. These prospects highlight the potential of the dataset to advance plant disease detection and precision agriculture research.

In the future, we plan to extend this dataset by publishing multispectral images acquired in controlled conditions for the same application. These images will integrate spatial and spectral information, providing a richer dataset for analysis. Combining spectral data with spatial context has the potential to significantly improve model performance by allowing the integration of complementary features. This dual-modality approach could facilitate the development of robust models capable of achieving highly accurate predictions, particularly for complex tasks such as distinguishing between similar symptoms or addressing spatially localized features.

The availability of both spectral and multispectral image data will not only support advanced research in grapevine disease detection, but also provide a basis for the development of practical tools for vineyard management. This comprehensive dataset represents a step forward in the use of spectral and imaging technologies to address key challenges in precision agriculture.

## Methods

### Sample Collection

The grapevine leaves were collected each September or October from 2020 to 2024, around the grape harvest period, on the Comité Champagne experimental site in Plumecoq, France. Those from 2020 to 2023 were collected after the harvest, while those for 2024 were collected before the harvest. This information is denoted by the “Year” variable in the dataset. For clarity thereafter, we use numerical codes to represent different years: 2x for 202x year. Table 1 provides an overview of the dates and temperatures recorded during each year’s data collection. Note that in 2020 and 2021 the temperatures were significantly higher, ranging from 20°C to 32°C, while in other years the temperatures were comparatively cooler, ranging from 10°C to 20°C. There was some rainfall in 2021. For these acquisitions, we waited until the leaves were partially dry, the remaining water being absorbed with paper towel. Note that for some acquisitions made in the morning, when there was morning dew, the same process was applied. Variations in temperature and humidity can affect spectral reflectance, particularly due to differences in humidity. Leaves exposed to cooler conditions tend to retain more moisture than those exposed to higher temperatures. Since spectral reflectance is fundamentally linked to molecular vibrations, increased leaf moisture could result in higher hydrogen content, potentially affecting spectral signatures.

**Table 1:** Every temperature from 2020 to 2024.

Year	Date	Temperature	Rain
2020	11 Sep	26°	no
	16 Sep	22°–32°	no
2021	07 Sep	22°–28°	no
	08 Sep	21°–28°	no
	09 Sep	21°–24°	yes(22mm)
	10 Sep	19°–22°	yes(13mm)
2022	22 Sep	13°–20°	no
	23 Sep	14°–19°	no
	26 Sep	12°	no
2023	02 Oct	19°–25°	no
	03 Oct	18°	no
	04 Oct	14°–17°	no
	05 Oct	14°–18°	no
2024	10 Sep	15°–18°	no
	11 Sep	13°	no
	12 Sep	12°–14°	no
	13 Sep	11°–16°	no

**Fig. 1:**
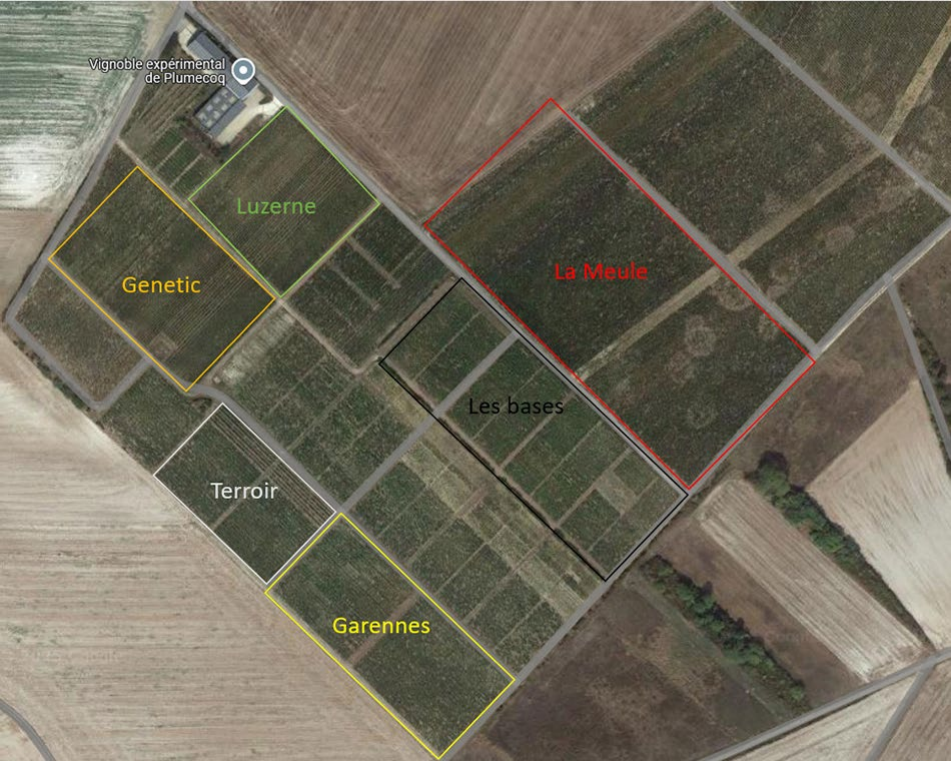
Fields of Chardonnay in Plumecoq.

The sampling was carried out in five areas, denoted by the variable “Zone” in the dataset, within Plumecoq domain: Garennes, Genetic, les Bases, Luzerne, and Terroir, but also in a neighboring plot belonging to a champagne producer, la Meule, which does not use the same treatments and has a more vigorous vine. During the years, the areas les Bases, Luzerne, and Terroir were introduced to increase the geographic diversity of the dataset. Figure 1 shows an aerial view of the Plumecoq vineyards and the neighboring plots. We indicated the six areas in the map with different colors, Our manipulation room is located in the gray building at the top of the image.

This study focused on five classes of Chardonnay vine leaves, denoted by the variable “Class” in the dataset: healthy, yellows (FD and BN), leafroll, esca, and discoloration. Note that discoloration is a new class observed since 2022. Vine classification were performed by experts from Comité Champagne through a prospecting process. Prospection involved walking through the vineyard plots to identify the five classes and label the corresponding plants accordingly. Table 2 gives an overview of the leaf samples collected annually from different classes in different regions of Plumecoq.

**Table 2:** Distribution of the Class variable over the Zone and Year variables.

	Garennes	Genetic	la Meule	les Bases	Luzerne	Terroir
Leafroll		20 to 24				
Esca	20 to,24	23	20 to 23		22,24	23,24
Yellows	20 to 24	20 to 23	20 to 24		22,23	24
Healthy	20 to 24	21,23	20 to 23		22 to 24	23,24
Discolorations	23,24	24	22,23	22	22 to 24	23

For each plant, we typically collected 16 leaves, with a few exceptions due to insufficient leaf availability. The leaves were selected from two height categories: apical height (A) and median height (M), with 8 leaves from each category. This information is denoted by the “Position” variable in the dataset.

### Spectra Acquisition

Once collected in the field, the leaves were quickly transported in plastic bags to the manipulation room, with a semi-controlled acquisition environment to ensure standardized measurement condition, spectra acquisitions being carried out in less than 20 minutes after collection.

**Fig. 2:**
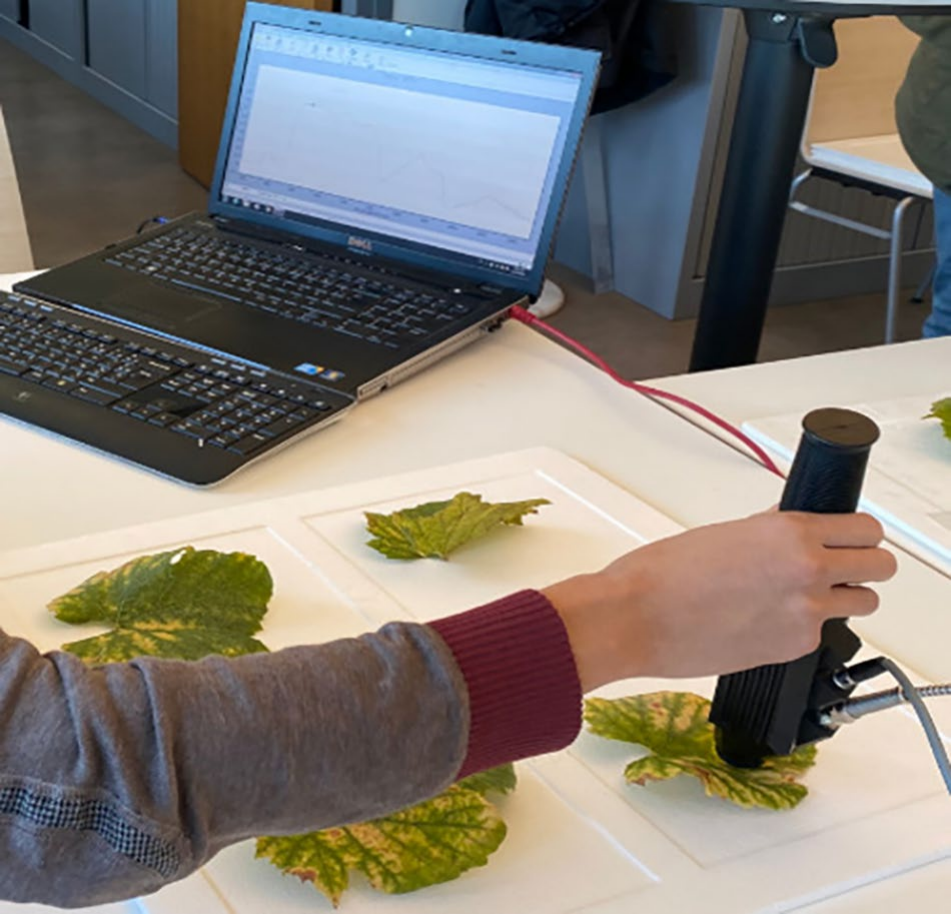
Spectra acquisition using the spectrometer and the probe.

Each leaf was placed on a white polystyrene plate and spectral data was acquired using a LabSpec® 4i ASD spectrometer (reference LS4Si-C2-C1-A0). This instrument offers a spectral resolution of 3 nm in the visible and near infrared and 10 nm in the shortwave infrared, making it well suited for accurate spectral analysis over a wide range of wavelengths, i.e. from 350 nm to 2500 nm. To minimize measurement errors caused by stray light, a contact probe was used for direct leaf measurements. The probe (refernce AK100500) has a fixed spot size of 10 mm, an operating length of 25.4 cm, and an integration time of 17 ms. The data acquisition software used was Indico Pro v 6.4.0. Each time the contact probe was placed on a leaf, a spectral measurement was recorded by averaging over 50 individual spectra, ensuring high accuracy and consistency (Fig. 2).

Spectra were collected from standardized positions on the leaf, typically the center and right side, to maintain uniformity across samples. This standardized approach ensured consistency across the dataset while allowing for variability in leaf morphology and disease presentation. Using the high-resolution capabilities of the LabSpec® 4i ASD spectrometer, we obtained accurate and detailed spectral data to facilitate robust analyses of disease-specific spectral signatures.

To ensure accurate spectral measurements, we first acquired a baseline spectrum on the white plate support before collecting the leaf spectra disposed on that plate, and this for each plate. This baseline allowed the spectrometer to pre-process the spectra in real time, making the measurements independent of the support material.

#### Acquisition in 2020

**Fig. 3:**
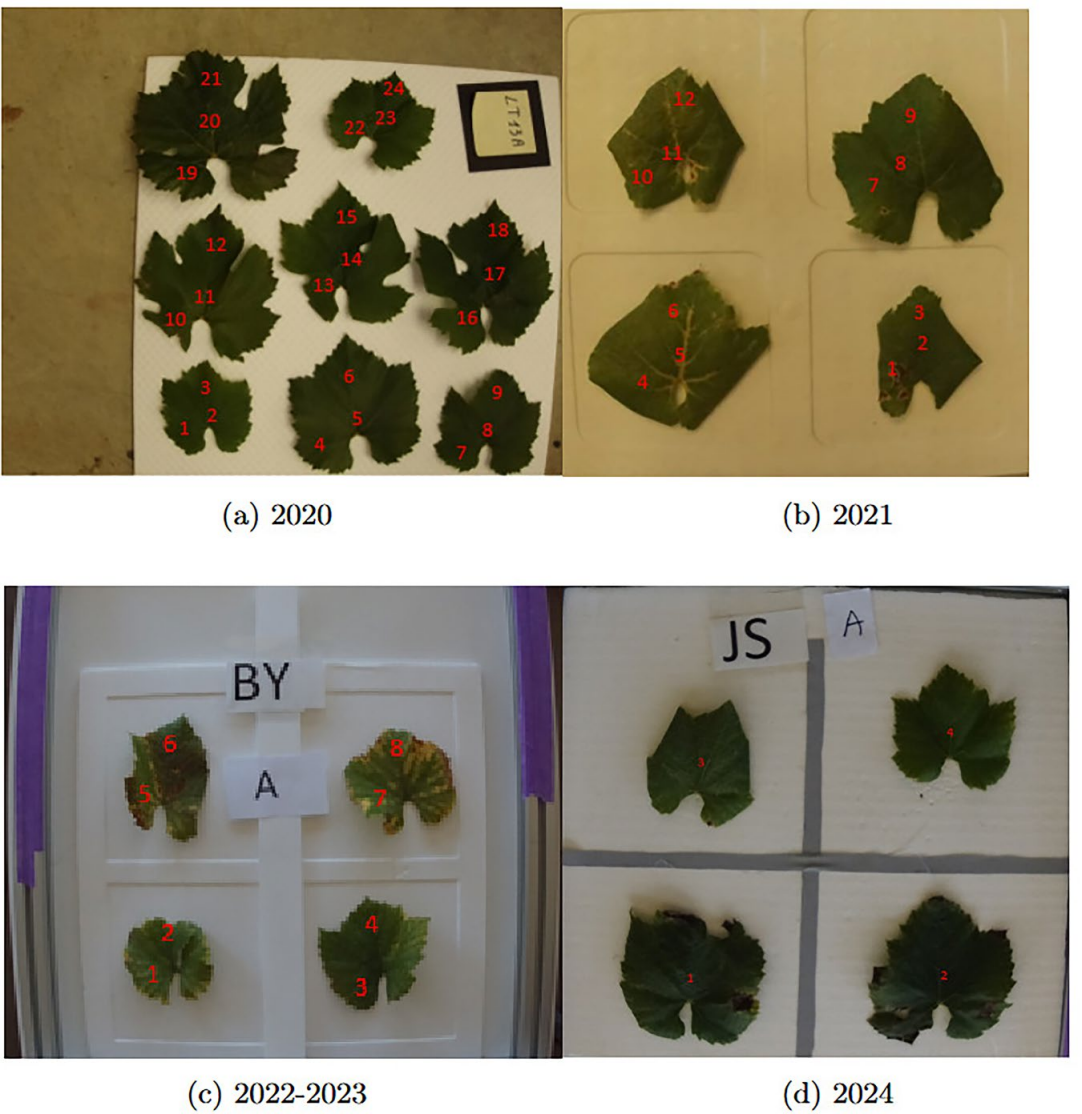
Location of acquisition zones of spectra on the leaves from 2020 to 2024.

In 2020, we put about 8 leaves on the white plate and the corresponding label, placed by the Comité Champagne on the vine indicating the Class, the Zone, and the Position variables, was placed in the corner. We took 3 spectra per leaf as shown in Fig. 3(a). Note that the state of health of the vineyards in Champagne in 2020 was very good. The vintage’s weather conditions resulted in very green and healthy foliage for the healthy class.

#### Acquisition in 2021

In 2021, we put the 4 leaves from the same position of each plant on a 4 × 4 grid on the same kind of white plate. The labels have been modified to indicate only the variable “Zone” and “Class”, coded by 2 letters, the “Position” variable being indicated by an additional label “A” or “M”. Initially, as we did in 2020, we acquired three spectra per leaf, as shown in Fig. 3(b). But as the acquisition progressed, we adjusted the protocol to collect two spectra per leaf instead, as shown in Fig. 3(c). Note that, in contrast to 2020, the health of the vineyards in Champagne in 2021 was severely damaged. Deteriorating weather conditions with heavy rainfall necessitated sustained phytosanitary protection, leaving a visible white layer on the foliage, particularly in the healthy class.

#### Acquisition in 2022 and 2023

In 2022, we introduced a new fifth class, the ‘discoloration’, for cases with confounding yellowish discoloration symptoms like manganese deficiency, chlorosis, magnesia deficiency, punctate, and symptoms linked to the buffalo leafhopper feeding ("Stictocephala bisonia”). The classes were designed to be balanced, but variability was observed during recording. Some symptoms were not always visible on the collected leaves, especially in yellows class, while others appeared as mixed symptoms, with yellows and esca on the same leaves. Note that the climate in Champagne in 2022 was very dry and hot before the harvest, with the leaves showing brown spots, particularly on the south-facing side of the row. In 2022 we took two spectra per leaf as shown in Fig. 3(c). The same 2 letters code was used to indicate the variables “Zone” and “Class”, and as previously an additional label “A” or “M” indicating the variable “Position”.

The acquisitions in 2023 were carried out under the same procedure. Note that, in 2022 and 2023, the state of health of the Champagne vineyards was healthy overall. The plots monitored were slightly affected by powdery mildew, which resulted in a phytosanitary protection that left visible white marks on the foliage.

#### Acquisition in 2024

The acquisitions in 2024 were carried out under the same procedure as in 2023, but we acquired only one spectrum per leaf as shown Fig. 3(d), however on a larger number of leaves. Note that, the Champagne vineyards were severely affected by downy mildew in 2024. As a result, the foliage of the plots monitored showed symptoms of leaf scorch, mostly at the top of the vines. A light white layer was also visible on the leaves of the healthy class due to the phytosanitary protection.

**Fig. 4:**
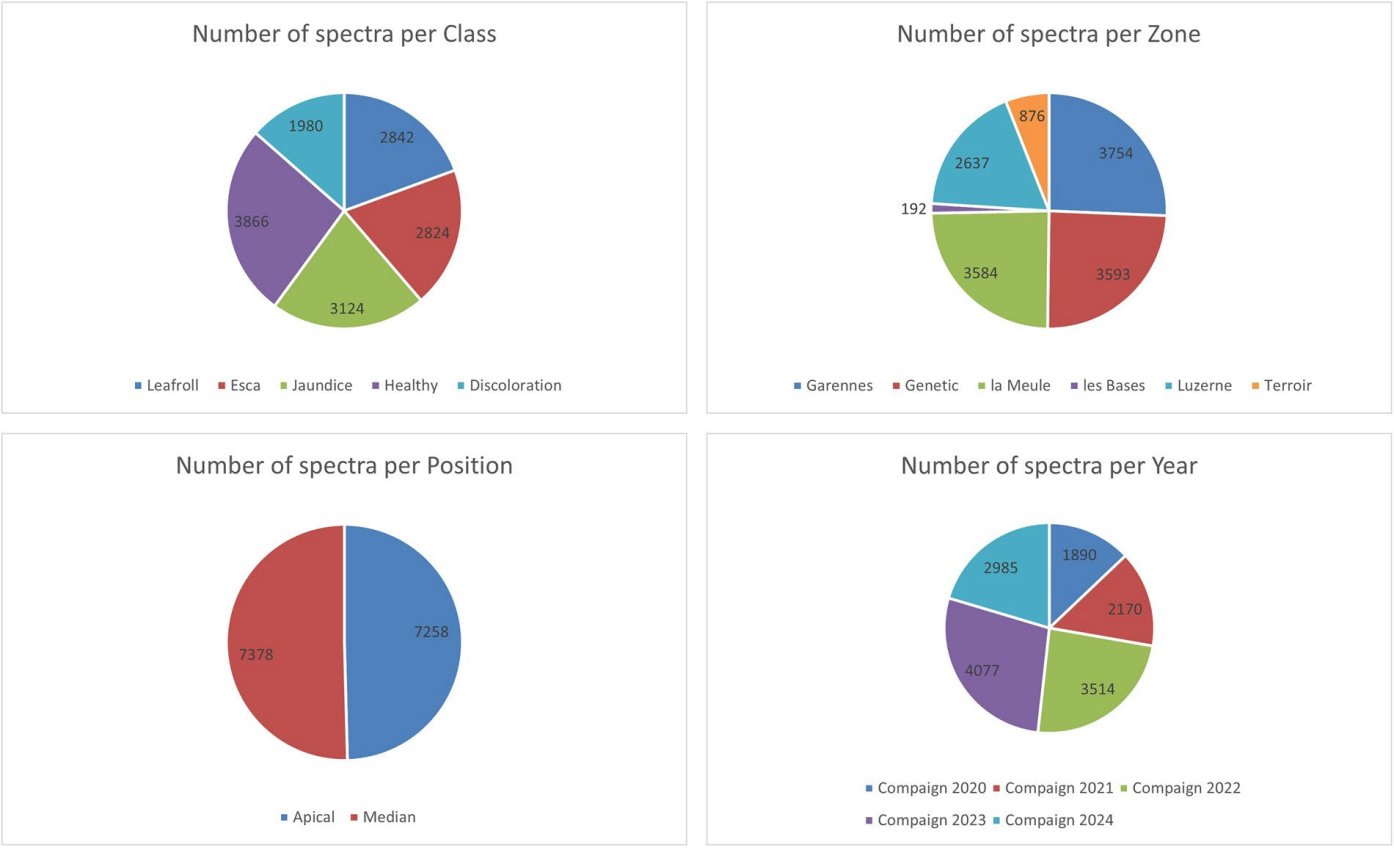
Number of spectra.

Over this 5 years acquisition campaign, 14636 spectra were collected. The values of the recorded reflectance spectral information during indicated in the dataset from 350 nm to 2500 nm every 1 nm. Figure 4 presents the distribution of spectra across the variables Class, Zone, Position, and Year. Regarding the class distribution, the number of spectra is relatively balanced across all classes, indicating no major class imbalance in the dataset. In terms of zones, a higher number of spectra were collected in the Garennes, Genetic, Luzerne and la Meule areas. For the leaf position, the number of spectra collected from the apical and median parts of the plants is comparable, ensuring consistent coverage across plant structures. Looking at the distribution by year, we observe a larger amount of data collected in 2022 and 2023. This increase is due to variations in the data acquisition protocol.

## Data Records

All spectra collected over the 5 years represents the rows of the dataset which is publicly available, in the form of an Excel table, on the Recherche Data Gouv^12^.

The ASD files collected by the Labspec® 4i spectrometer were converted into ASCII files containing reflectance spectral information, with none derivative, using the Indico Pro v 6.4.0 software. Each spectrum correspond to a line, with columns from A to CDS containing the reflectance spectral information every 1nm from 350 to 2500 nm.

Text labels corresponding to class, regions, and heights were transformed into numeric data and added as numeric information at the end of each line, along with the year of acquisition:

- The five classes are coded on the “Class” column CDT by: 0 for Leafroll, 1 for Esca, 2 for Yellows, 3 for Healthy, and 4 for Discoloration.
- The six different areas are coded on the “Zone” column CDU by: 0 for Garennes, 1 for Genetic, 2 for la Meule, 3 for les Bases, 4 for Luzerne, and 5 for Terroir.
- The two height categories are coded on the “Position” column CDV by: 0 for apical height leaves A and 1 for median height leaves M.
- The five acquisition years are represented by a numerical value in the “Year” column CDW, where 2020 is coded 0, 2021 is coded 1, 2022 is coded 2, 2023 is coded 3, and 2024 is coded 4.

## Technical Validation

### Preprocessing Techniques for Spectral Data Analysis

Preprocessing spectral data is often essential for the analysis of infrared spectra. By minimizing the influence of external factors such as noise and scatter effects, preprocessing enhances the reliability and comparability of spectral datasets.

Let ${S}_{k}[{x}_{i}]\in {{\mathbb{R}}}^{n}$ be the reflectance of the *k*^*th*^ acqusition for the wavelength *x*_*i*_ in the range [*x*_1_, …, *x*_*n*_], with *x*_1_ = 350 nm, *x*_*n*_ = 2500 nm, and *n* = 2151. The following techniques could be used to preprocess the data:

**L1 or L2 Normalization** Normalization techniques such as L1 and L2^13^ are commonly used in spectral preprocessing to correct for magnitude differences and improve comparability between samples. These methods scale each spectral vector *x* to a standard reference form, but differ in their scaling criteria.

L1 normalization scales the spectral vector so that the sum of the absolute values equals one:

1 $${S}_{k}^{{\prime} }[{x}_{i}]=\frac{{S}_{k}[{x}_{i}]}{{\sum }_{j=1}^{n}| {S}_{k}[{x}_{j}]| }.$$

This simple and fast to compute approach emphasizes the relative contribution of each wavelength, making it useful in contexts where sparsity or proportionality is important. However, it can distort the shape of the spectrum if intensity differences are meaningful, being also sensitive to outliers.

L2 normalization, used in this study, scales vectors based on their Euclidean norm:

2 $${S}_{k}^{{\prime} }[{x}_{i}]=\frac{{S}_{k}[{x}_{i}]}{\sqrt{{\sum }_{j=1}^{n}{S}_{k}{[{x}_{j}]}^{2}}}.$$

By normalizing each spectrum to unit length, L2 normalization preserves directional information (i.e., the shape of the spectral signature) while eliminating magnitude differences caused by external factors such as illumination intensity, sensor variability, or sample thickness, ensuring all spectra are on a similar scale. More robust to outliers than L1, can be however sensitive to spectral peaks with high intensities and may distort the relative peak heights. L2 normalization is particularly useful for machine learning and multivariate analysis where consistent scaling across samples is critical. It ensures that all spectra lie on the surface of a hypersphere in feature space, which can improve convergence and stability in algorithms sensitive to feature scale, such as distance-based classifiers or neural networks.

Although L1 and L2 are standard in the literature, several other normalization and preprocessing techniques-such as Standard Normal Variate (SNV), Savitzky-Golay (SG) smoothing and derivatives, and Multiplicative Scatter Correction (MSC)-also play an important role in spectral data enhancement.

**The Standard Normal Variate (SNV)**^14,15^ transformation can be applied to mitigate the effects of scattering and multiplicative interference caused by surface irregularities or particle size. This technique adjusts each spectral vector by centering and scaling it. Each element of the vector is transformed as follows:

3 $${S}_{k}^{{\prime} }[{x}_{i}]=\frac{{S}_{k}[{x}_{i}]-\mu }{\sigma },$$

where *μ* is the mean of *S*_*k*_ and *σ* is the standard deviation of *S*_*k*_ after subtracting the mean.

SNV improves the comparability of spectra between samples, effectively reducing baseline shifts and variations within spectra. Being useful for comparing spectra where relative intensity is important, is however sensitive to outliers and assumes that the spectrum is normally distributed.

**Multiplicative Scatter Correction (MSC)**^16^ compensates for additive and multiplicative effects in spectral data due to scattering and surface variability. Each spectrum is adjusted based on a reference spectrum *R*[*x*_*i*_], typically the mean spectrum of all samples, i.e. $R[{x}_{i}]=\frac{1}{K}{\sum }_{k=1}^{K}{S}_{k}[{x}_{i}]$, with *k* the number of available spectra. MSC performs a linear regression between each spectrum *S*_*k*_[*x*_*i*_] and the reference spectrum, i.e. *S*_*k*_[*x*_*i*_] = *a*_*i*_ + *b*_*i*_*R*[*x*_*i*_] + *ϵ* to estimate the offset *a*_*i*_ and slope *b*_*i*_, then corrects as:

4 $${S}_{k}^{{\prime} }[{x}_{i}]=\frac{{S}_{k}[{x}_{i}]-{a}_{i}}{{b}_{i}}.$$

This normalization aligns the spectra to a common baseline and removes the multiplicative effects, improving comparability and the robustness of downstream multivariate analyses. However, it requires a reference spectrum, which might not always be available, and can distort data if this reference is not representative of all samples.

**The Savitzky-Golay (SG) filter**^17^ introduced by Savitzky and Golay, is a widely used smoothing technique in spectral analysis. Unlike simple moving averages, the SG filter preserves the shape and height of spectral features, making it particularly suitable for applications in chemometrics and remote sensing.

The key idea behind the SG filter is that smoothing can be achieved through local polynomial regression. Specifically, for each point in the spectrum, a degree polynomial *p* is fitted to a symmetric window of neighboring points *k* = 2*m* + 1 (where *m* is the size of the half-window). The value of the polynomial in the center of this window is taken as the smoothed value.

The smooth value of SG is

5 $${S}_{k}^{{\prime} }({x}_{i})={P}_{I}({S}_{k}[{x}_{i}]),$$

where *P*_*I*_ is the polynomial fitted locally to the interval *I* = [*x*_*i*−*m*_, …, *x*_*i*+*m*_].

The SG filter effectively computes a weighted sum of the spectral points within the window, where the weights (or convolution coefficients) are derived from the least squares fit of the polynomial. These coefficients are fixed for a given *k* and *p* and can be precalculated to improve efficiency.

One of the major advantages of the SG filter is that it also supports the calculation of derivatives, which can be used to highlight changes in slope and curvature in the spectrum, features that are useful for identifying subtle absorption peaks or inflection points. The first and second derivatives are given by

6 $${S}_{k}^{(1)}({x}_{i})={P}_{I}^{(1)}({S}_{k}[{x}_{i}]),\quad {S}_{k}^{(2)}({x}_{i})={P}_{I}^{(2)}({S}_{k}[{x}_{i}]),$$

where ${P}_{I}^{(1)}$ and ${P}_{I}^{(2)}$ are the first and second derivatives of the locally fit polynomial *P*_*I*_.

A larger window size *k* smooths more aggressively, which reduces noise, but may distort narrow spectral features or peaks. A higher degree polynomial *p* can model more complex local behavior, but can also lead to overfitting, especially if *p* approaches *k*. It is thus important to choose *p* and *k* carefully based on the nature of the data and the application. For noisy spectra with fine detail, a smaller *k* and a moderate *p* often provide a good balance between noise reduction and feature preservation.

### Analysis of average spectra

In this section, we applied L2 normalization prior to analyzing the average spectra. The average spectrum was then computed for each categorical variable: class, zone, position, and year.

**Fig. 5:**
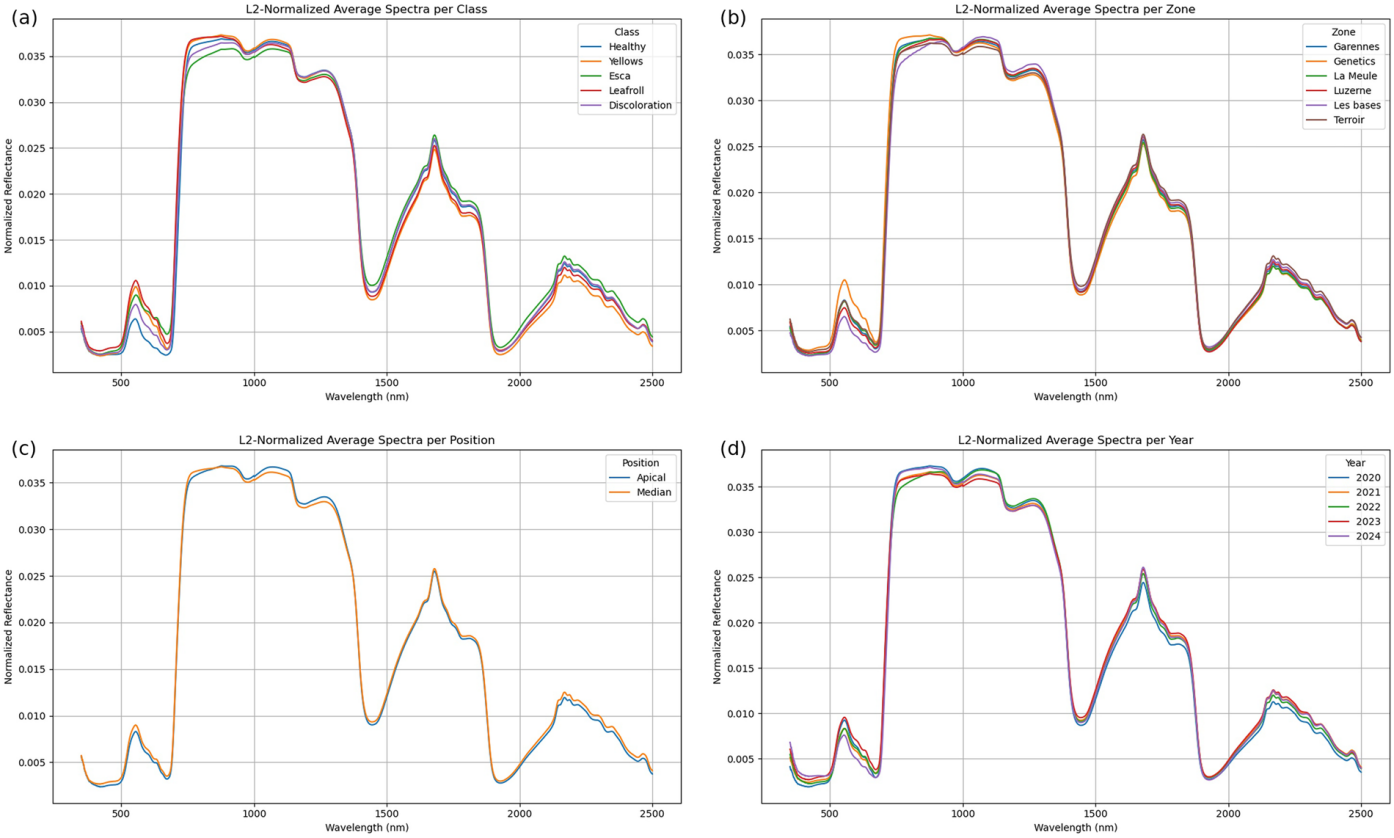
Analysis of L2-norm average spectra.

The Fig. 5(a) presents these average spectra, highlighting subtle but meaningful differences across several categories. For the class-level analysis, noticeable variations are observed around 550 nm, 730 nm, and 2200 nm. These wavelengths may serve as key discriminative bands for class separation, suggesting their potential importance in classification tasks.

In terms of zone-based differences, the bands near 550 nm and 730 nm continue to show discriminatory power, particularly in distinguishing the Genetics zone from the others, as shown in Fig. 5(b). The Genetics variety shows a clearer separation from other zones in these spectral regions, indicating a unique spectral signature. This result was expected given the specificity of grape varieties in this region.

When spectra are compared on the basis of position (apical vs. median), the average spectra appear quite similar and show no significant differences, as shown in Fig. 5(c). This suggests that the position of the leaf does not strongly influence the spectral variation in the L2 normalized space, at least around the grape harvest period.

Finally, the annual comparison shows slight differences, especially around 730 nm, as shown in Fig. 5(d). This variation could be influenced by environmental factors which vary between growing seasons and can affect spectral reflectance. As highlighted, due to the climatic conditions and phytosanitary treatments depending on the year, the leaves do not have the same visual appearance, their color being affected.

Simply observing differences in average spectra may not be sufficient to identify the most relevant discriminative bands, especially for distinguishing the Yellows class, which shows significant similarities to the Esca and Leafroll classes. It should be noted that our previous work^10,11^ also identified the 550 nm and 730 nm bands through advanced band selection techniques, but also other bands, further reinforcing their importance in disease classification.

### Principal Component Analysis (PCA)

Since our spectral data consists of thousands of wavelength bands, resulting in high-dimensional data, Principal Component Analysis (PCA) is an effective technique for reducing this dimensionality while preserving most of the variance in the data. This makes analysis and visualization easier^18^.

PCA is a dimensionality reduction technique that transforms high-dimensional spectral data into a new coordinate system. In this system, each axis corresponds to a principal component (PC), a direction in the feature space along which the data vary the most. Each PC is a linear combination of the original spectral bands, and the loadings (i.e., the coefficients of that combination) represent the contribution of each original band to that component. A high absolute loading for a band indicates that it strongly influences the corresponding PC, meaning that the band plays a significant role in capturing variance along that direction. Conversely, low absolute loadings indicate that the band has little influence on that PC. By examining the loadings, one can interpret which spectral regions are most important in explaining variability in the data, making PCA useful not only for compression, but also for identifying informative wavelengths.

**Fig. 6:**
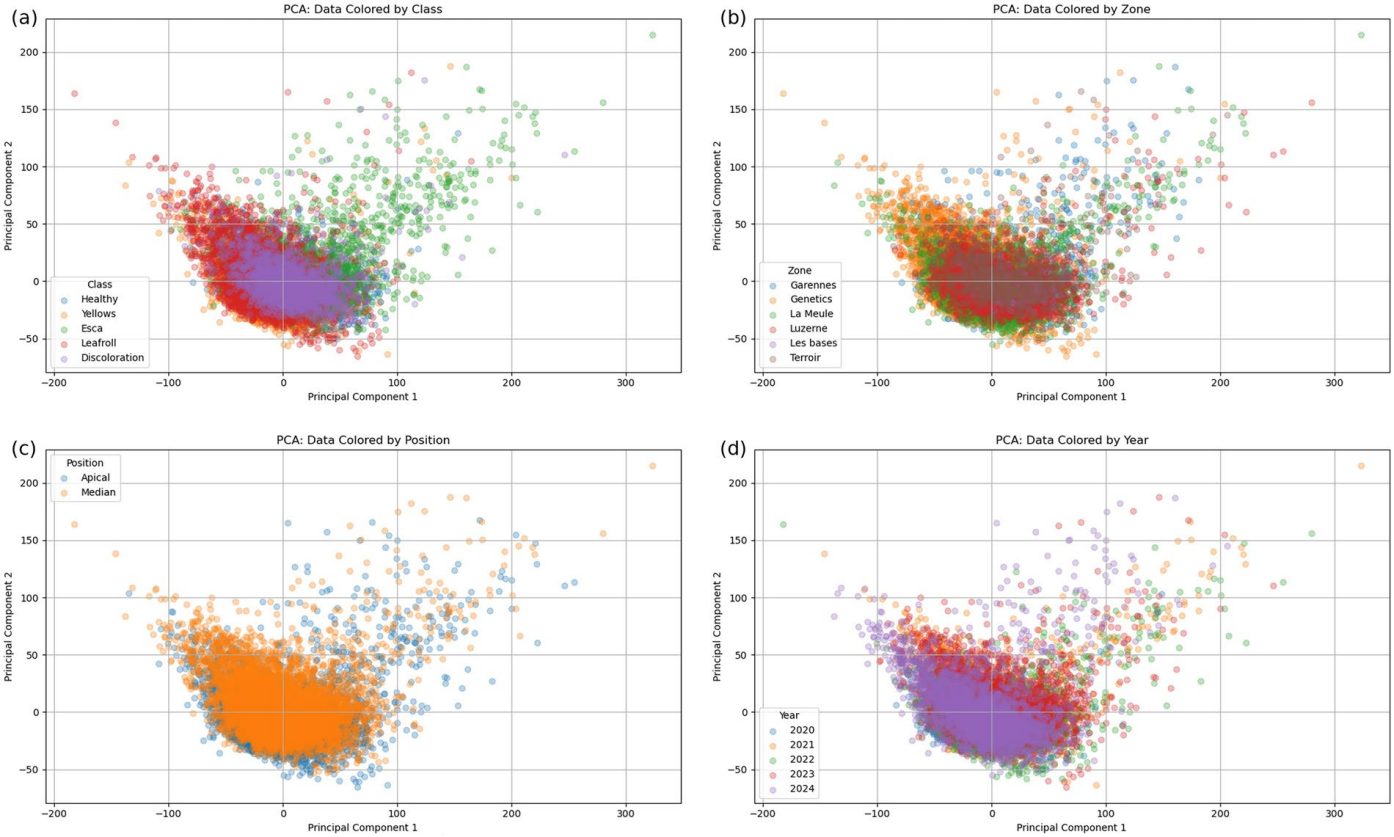
PCA of spectra.

Figure 6 shows the PCA visualization of the spectral data across the four variables: class, zone, position, and year. Regardless of the label category, the separation between groups appears to be limited. This result is expected given the high degree of spectral similarity among the varieties. The PCA projections show overlapping clusters, indicating that this linear dimensionality reduction technique struggles to effectively discriminate between the spectra. This highlights the inherent difficulty of class separation based solely on spectral data using PCA.

**Fig. 7:**
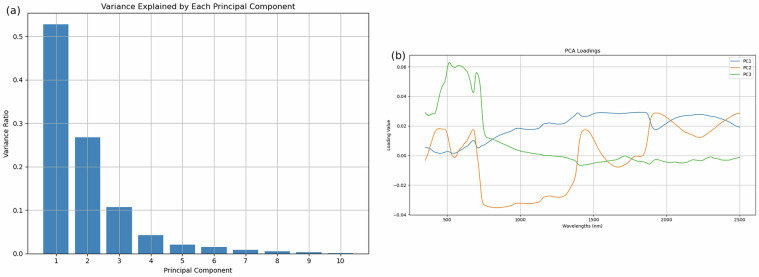
Analysis of PCA.

The bar plot in Fig. 7(a) displays the proportion of the total variance captured by each PC. Principal Component 1 (PC1) accounts for more than 50% of the variance, while PC2 explains about 27% and PC3 contributes about 11%. Together, the first three PCs account for approximately 88% of the total variance, indicating that they capture the most significant patterns in the data set. Beyond PC3, the variance explained by each component drops sharply and becomes almost negligible. This result suggests that dimensionality reduction is highly effective for our spectral data. By retaining only the first two or three PCs, we preserve most of the information while reducing noise and computational complexity.

The line plot of the PCA loadings in Fig. 7(b) shows how each original wavelength contributes to the first three PCs. These loadings provide insight into the underlying spectral features associated with each PC, reflecting important physiological and biochemical characteristics of the vegetation.

- PC1 (blue line) shows strong and relatively stable contributions throughout the mid- to near-infrared region (1000-2500 nm), with notable peaks at 1400 nm, 1900 nm, and 2200 nm. These wavelengths are known absorption features for water, cellulose, lignin, and protein content: The 1400 and 1900 nm bands are sensitive to leaf water content due to strong water absorption^19,20^. And the 2200 nm region is associated with cellulose, lignin, and protein absorption^21,22^. These results suggest that PC1 reflects overall tissue structure and moisture content.
- PC2 (orange line) displays greater variation, with prominent peaks in the visible (400-700 nm) and again in the shortwave infrared (SWIR) (1900-2500 nm). This component likely captures pigment-related changes and structural stress: The visible region corresponds to the absorption of chlorophyll a and b (especially near 430 nm and 660 nm), which are critical for photosynthetic activity^23^. Peaks in the SWIR also correspond to cell wall structure and biochemical content, possibly reflecting stress-induced changes in leaf composition.
- PC3 (green line), while active in a similar visible range, typically carries finer, more nuanced variations. The variability explained is minimal (around 11%), suggesting that it is more likely to accentuate nuanced features such as early chlorosis, pigment imbalance, or local discoloration. These features, while potentially less dominant in the overall signal, are nevertheless significant for specific detection tasks, particularly in the context of early-stage disease.

## Data Availability

All data supporting this study are available on Recherche Data Gouv, 10.57745/KPNOJL^12^. The deposit includes leaf reflectance spectra (350–2500 nm), metadata (year, zone, leaf position, and class labels), and acquisition documentation. There are no access restrictions.

## Code availability

The Python code is provided as a Jupyter Notebook within the project workspace (see https://github.com/zsr1997/Scientific-Data). It provides a step-by-step guide for accessing, visualizing, and analyzing the spectral data set. The notebook includes visualization of all spectral measurements and generation of average spectra per class in different years. These resources are designed to promote reproducibility and support further exploration of the dataset and analysis methods.

## References

[1] Chuche, J. & Thiéry, D. Biology and ecology of the *flavescence dorée* vector *Scaphoideus titanus*: A review. *Agronomy for Sustainable Development* **34**, 381–403, 10.1007/s13593-014-0208-7 (2014).

[2] Tessitori, M., La Rosa, R. & Marzachí, C. Flavescence Dorée and Bois Noir Diseases of Grapevine Are Evolving Pathosystems. *Plant Health Progress* **19**(2), 136–138, 10.1094/PHP-10-17-0057-MR (2018).

[3] Naidu, R. A., Perry, E. M., Pierce, F. J. & Mekuria, T. The potential of spectral reflectance technique for the detection of grapevine leafroll-associated virus-3 in two red-berried wine grape cultivars. *Computers and Electronics in Agriculture* **66**, 38–45, 10.1016/j.compag.2008.11.007 (2009).

[4] Hou, J., Longtang, L. & Jie, H. Detection of grapevine leafroll disease based on 11-index imagery and ant colony clustering algorithm. *Precision Agriculture*, 17, 10.1007/s11119-016-9432-2 (2016).

[5] Owomugisha, G., Nuwamanya, E., Quinn, J. A., Biehl, M. & Mwebaze, E. Early detection of plant diseases using spectral data. In *Proceedings of the 3rd International Conference on Applications of Intelligent Systems (APPIS 2020)*, Association for Computing Machinery, New York, NY, USA, 10.1145/3378184.3378222 (2020).

[6] Belasque, J., Gasparoto, M. C. & Marcassa, L. G. Detection of mechanical and disease stresses in citrus plants by fluorescence spectroscopy. *Applied Optics* **47**, 1922–1926, 10.1364/AO.47.001922 (2008). 18404192 10.1364/ao.47.001922

[7] AL-Saddik, H., Simon, J.-C. & Cointault, F. Development of spectral disease indices for ‘flavescence dorée’ grapevine disease identification. *Sensors*, 17, 10.3390/s17122772 (2017). 10.3390/s17122772 PMC5751714 29186057

[8] Thielert, B. *et al*. Phenotruckai: On-site hyperspectral measurement for distinction of quarantine grapevine disease “flavescence dorée” and non-quarantine disease “bois noir” in a mobile laboratory. In *INFORMATIK 2024*, pages 1253–1260. Gesellschaft für Informatik e.V., Bonn, 10.18420/inf2024_110 (2024).

[9] Carli, M. *et al*. Using hyperspectral data to early detect “flavescence dorée” in Tuscany vineyards. In *FD2024 Book of Abstracts*. University of Pisa, University of Florence, University of Salento, Regional Phytosanitary Service, (2023). (Note: Study in Tuscany on early detection of FD using hyperspectral data.)

[10] Zhang, S., Goupil, A., Vrabie, V., Perrin, E. & Panon, M.-L. Multispectral band selection using correlation explanation for identification of discriminative bands related to grapevine diseases. In *2024 IEEE Information Theory Workshop (ITW)*, pages 502–507, 10.1109/ITW61385.2024.10806927 (2024).

[11] Zhang, S., Goupil, A., Vrabie, V., Perrin, E. & Panon, M.-L. A bottom-up approach to select constrained spectral bands discriminating vine diseases. In *2024 19th Conference on Computer Science and Intelligence Systems (FedCSIS)*, pages 707–712, 10.15439/2024F7286 (2024).

[12] Zhang, S., Perrin, E., Vrabie, V., Goupil, A. & Panon, M.-L. Multi-annual spectral data of Chardonnay grapevine leaves, 10.57745/KPNOJL (2025).

[13] Pedregosa, F. *et al*. Scikit-learn: Machine learning in Python, 10.48550/arXiv.1201.0490 (2018).

[14] Luypaert, J., Heuerding, S., Heyden, Y. V. & Massart, D. The effect of preprocessing methods in reducing interfering variability from near-infrared measurements of creams. *Journal of Pharmaceutical and Biomedical Analysis* **36**, 495–503, 10.1016/j.jpba.2004.06.023 (2004). 15522523 10.1016/j.jpba.2004.06.023

[15] Barnes, R. J., Dhanoa, M. S. & Lister, S. J. Standard normal variate transformation and de-trending of near-infrared diffuse reflectance spectra. *Applied Spectroscopy* **43**, 772–777, 10.1366/0003702894202201 (1989).

[16] Geladi, P., MacDougall, D. & Martens, H. Linearization and scatter-correction for near-infrared reflectance spectra of meat. *Applied Spectroscopy* **39**, 491–500, 10.1366/0003702854248656 (1985).

[17] Savitzky, A. & Golay, M. J. E. Smoothing and differentiation of data by simplified least squares procedures. *Analytical Chemistry* **36**, 1627–1639, 10.1021/ac60214a047 (1964).

[18] Jolliffe, I. T. & Cadima, J. Principal component analysis: a review and recent developments. *Philosophical Transactions of the Royal Society A: Mathematical, Physical and Engineering Sciences* **374**, 20150202, 10.1098/rsta.2015.0202 (2016). 10.1098/rsta.2015.0202 PMC4792409 26953178

[19] Carter, G. A. Primary and secondary effects of water content on the spectral reflectance of leaves. *American Journal of Botany* **78**, 916–924, 10.1002/j.1537-2197.1991.tb14495.x (1991).

[20] Gao, B.-C. NDWI — a normalized difference water index for remote sensing of vegetation liquid water from space. *Remote Sensing of Environment* **58**, 257–266, 10.1016/S0034-4257(96)00067-3 (1996).

[21] Curran, P. J. Remote sensing of foliar chemistry. *Remote Sensing of Environment* **30**, 271–278, 10.1016/0034-4257(89)90069-2 (1989).

[22] Kokaly, R. F. & Clark, R. N. Spectroscopic determination of leaf biochemistry using band-depth analysis of absorption features and stepwise multiple linear regression. *Remote Sensing of Environment* **67**, 267–287, 10.1016/S0034-4257(98)00084-4 (1999).

[23] Gitelson, A. A., Gritz, Y. & Merzlyak, M. N. Relationships between leaf chlorophyll content and spectral reflectance and algorithms for non-destructive chlorophyll assessment in higher plant leaves. *Journal of Plant Physiology* **160**, 271–282, 10.1078/0176-1617-00887 (2002). 10.1078/0176-1617-00887 12749084

